# First and Second Metatarsophalangeal Joint Open Dislocations: A Case Report

**DOI:** 10.5704/MOJ.1703.010

**Published:** 2017-03

**Authors:** A Sharma, R Dosajh, GS Bedi, K Gupta, A Jain

**Affiliations:** Department of Orthopaedics, Government Multispecialty Hospital, Chandigarh, India

**Keywords:** metarso-phalangeal, joint, dislocation

## Abstract

Dislocation of multiple metatarsophalangeal joint is an uncommon injury. The mechanism of injury is a high energy force distal to proximal with foot in hyperextension at the metatarsophalangeal (MTP) joint. The acute hyperextension of the toe at the moment of injury causes avulsion of the plantar part of the capsule from the junction of head and neck of the metatarsal. If the collateral ligaments remain intact, they maintain the locked fibrocartilaginous plate over the dorsum of the head of the metatarsal, making closed reduction impossible. We report a case of simultaneous 1st and 2nd MTP joint open dislocation. In the present case, we chose the plantar approach utilizing the already present plantar wound. At 18 months post-operative follow-up, there was no instance of redislocations or signs of avascular necrosis of head of metatarsal.

## Introduction

Dislocation of multiple metatarsophalangeal joint is an uncommon injury. However, if left untreated or improperly managed, such an injury can have a deleterious effect on patient’s ability to bear weight and walk. Motor vehicle accidents, followed by fall from height and athletic injuries, are the most common causes of this injury^1^. The mechanism of injury is a high energy force distal to proximal with foot in hyperextension at the MTP joint. Jahss classified dorsal dislocations of the first metatarsophalangeal joint into three types according to the disruption of the sesamoid mass^2^ (Table I). The importance in classifying these injuries is that it allows one to predict whether or not closed reduction will be successful. The acute hyperextension of the toe at the moment of injury causes avulsion of the plantar part of the capsule from the junction of head and neck of the metatarsal. This allows the base of the proximal phalanx to slide over the metatarsal head followed by the fibrocartilaginous plate and becomes locked in this position. If the collateral ligaments remain intact, they maintain the locked fibrocartilaginous plate over the dorsum of the head of the metatarsal, making closed reduction impossible. We report a case of simultaneous open dislocation of the 1st and 2nd MTP joints. Only two such cases have been reported in the literature, to the best of our knowledge^3^,^4^.

**Table I:** Jahss Classification System of first Metatarsophalangeal Joint Dislocations

Stage	Type of dislocation
Type I	Dorsal dislocation without disruption of sesamoid complex. Usually not reducible closed.
Type IIA	Dislocation with longitudinal disruption of volar plate and intersesamoid ligament, noted by increased distance between sesamoids.
Type IIB	Partial disruption of volar plate with disruption of either medial or lateral sesamoid.
Type III	Complete soft tissue disruption of the volar complex from the proximal phalanx.

## Case Report

**Fig. 1:**
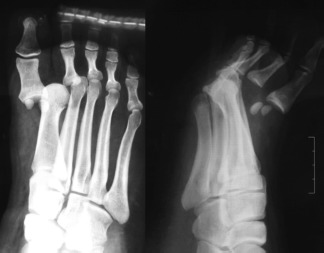
Preoperative radiographs AP and lateral views.

We report the case of a healthy 19-year old male with no significant past medical or surgical history who injured his right foot his right foot in a road traffic accident and reported to our hospital within two hours of the injury. On examination, there was significant swelling of the foot with a 1.5 cm x 1 cm wound on the plantar aspect through which a bony prominence that appeared to be the head of the first metatarsal was visible. Sensation and vascularity of the foot were normal. Radiographs of the injured foot showed dorsal dislocation of the first and second MTP joints with dorsal displacement of the sesamoid complex (Fig. 1).

After thorough irrigation and debridement of the wound under local anesthesia, reduction was attempted within four hours of the injury using longitudinal traction and dorsally directed pressure over head of metatarsal through the wound and simultaneous plantar directed force on base of proximal phalanx in hyperextension as well as neutral position of the MTP joint, but reduction was not achieved. Patient was then investigated further and in the emergency OT under spinal anesthesia around 12 hours after the injury, reduction was again attempted but failed. The plantar wound (approx 2cm x 1.5 cm in size) which was already located in a nearly transverse direction over the 1st and 2nd metatarsal heads was extended 1.5 cm distally and medially for adequate exposure.

**Fig. 2:**
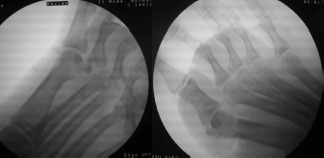
Intraoperative radiograph after reduction of first and then both MTP joint dislocation.

**Fig. 3:**
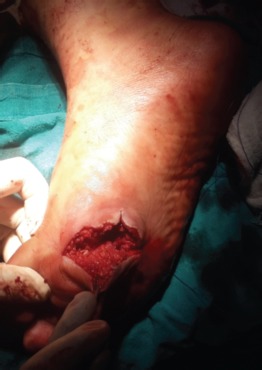
Plantar wound after reduction of dislocation.

The head of the first metatarsal was found to be protruding plantarwards through a rent in the plantar fascia with intact collateral ligaments. The lateral collateral ligament was divided, a lever placed dorsal to the proximal phalanx, longitudinal traction and dorsal push was applied to the head of the metatarsal to achieve reduction of the 1st MTP joint. The sesamoid complex followed the proximal phalanx but the 2nd MTP joint remained unreduced after an attempt at closed reduction. A lever was applied dorsal to the head of 2nd metatarsal along with longitudinal traction through the same wound to facilitate its leverage out of deep transverse metatarsal ligament lying dorsal to its neck to achieve reduction. The reduction of both the MTP joints was assessed for stability after repair of the divided collateral ligament and there was no requirement for fixation with K-wires. (Fig. 2, Fig. 3).

The wound was closed in layers after thorough irrigation and debridement and a bulky dressing with a below knee POP splint was applied. Immediate post-operative radiographs showed proper position and alignment of the joints and sessamoid apparatus. The post-operative dressing was clean and dry and there was no neurovascular deficit. Patient was discharged and followed up for dressings. and was kept nonweight bearing. The wound had healed well, with minimal oedema, and sutures were removed on the 14th postoperative day, and the patient was able to actively plantar flex and dorsiflex the MTP joints.

The extremity was placed in a walking cast boot with toe-touch weight bearing aided with a stick for two weeks and then the patient was instructed to bear weight as tolerated on the right foot and advised active and passive range of motion exercises. At one month follow up post-operatively, the patient had good active and passive range of motion and had only mild pain on excessive walking. Thereafter, the patient was evaluated monthly for six months, then three monthly and 12 months and 18 months post-operative follow-up. There was no instance of redislocations or signs of avascular necrosis of the heads of the metatarsals. Range of motion was similar to the contralateral foot and walking was pain free.

## Discussion

Traumatic dislocations of the metatarsophalangeal joints are rare injuries, with very few reports of simultaneous MTP joint dislocation of 1st and 2nd toes. The capsule of the 1st MTP joint has several reinforcements that contribute to the joint stability: the plantar plate, medial and lateral collateral ligaments, tendons of the flexor hallucis longus and brevis, adductor and abductor hallucis, and the extensor hallucis longus and brevis^3^. The joint is relatively unprotected on the dorsal side, making the dorsal dislocation more common. Once dislocated, these structures interfere with the reduction of the joint. Similarly, reduction of the other MTP joints is interfered because the deep transverse metatarsal ligament and plantar plate are strong and are caught by the dorsal side of the metatarsal neck or due to obstruction by the flexor tendons or lumbricals independently or in combination.

Open reduction should be performed when manipulative reduction fails. However, in most instances dorsal approaches have been used previously for open reduction of dorsal dislocation leading to the incised deep transverse metatarsal ligament and plantar plate being unrepaired, thus requiring K-wires for stability. Therefore, Nakano *et al*^5^ suggested performing the plantar approach to facilitate reduction as well as repair of the plantar plate and deep transverse metatarsal ligament. Possible limitations of this approach include injury to the plantar neurovascular structures resulting in a painful sensitive scar on the weightbearing aspect of the metatarsal pad. Therefore, meticulous dissection is required with utilizing a curved transverse incision over the metatarsal heads area.

In the present case, we chose the plantar approach utilizing the already present plantar wound. As a result, we could look at the obstruction directly, and satisfactory stability was achieved by strongly re-suturing the collateral and deep transverse metatarsal ligaments. Moreover, reduction of the first MTP dislocation did not lead to simultaneous reduction of second MTP dislocation, as occurs in most instances of multiple MPT dislocations of the other toes, thus enforcing the point that the structures involved in impediment of closed reduction of both these joints are anatomically different.
